# Acceptance and User Experiences of a Wearable Device for the Management of Hospitalized Patients in COVID-19–Designated Wards in Ho Chi Minh City, Vietnam: Action Learning Project

**DOI:** 10.2196/44619

**Published:** 2024-01-05

**Authors:** An Phuoc Luu, Truong Thanh Nguyen, Van Thi Cam Cao, Trinh Hoang Diem Ha, Lien Thi Thu Chung, Trung Ngoc Truong, Tung Nguyen Le Nhu, Khoa Bach Dao, Hao Van Nguyen, Phan Nguyen Quoc Khanh, Khanh Thuy Thuy Le, Luu Hoai Bao Tran, Phung Tran Huy Nhat, Duc Minh Tran, Yen Minh Lam, Catherine Louise Thwaites, Jacob Mcknight, Nguyen Van Vinh Chau, Jennifer Ilo Van Nuil, Vietnam ICU Translational Applications Laboratory (VITAL)

**Affiliations:** 1 Oxford University Clinical Research Unit Ho Chi Minh City Vietnam; 2 Hospital for Tropical Diseases Ho Chi Minh City Vietnam; 3 Centre for Tropical Medicine and Global Health Nuffleld Department of Medicine University of Oxford Oxford United Kingdom; 4 See Acknowledgments

**Keywords:** vital signs, wearable devices, action learning, technology acceptance model, TAM, COVID-19, user-centered design, wearables, remote monitoring, technology acceptance, oximeter

## Abstract

**Background:**

Wearable devices have been used extensively both inside and outside of the hospital setting. During the COVID-19 pandemic, in some contexts, there was an increased need to remotely monitor pulse and saturated oxygen for patients due to the lack of staff and bedside monitors.

**Objective:**

A prototype of a remote monitoring system using wearable pulse oximeter devices was implemented at the Hospital for Tropical Diseases in Ho Chi Minh City, Vietnam, from August to December 2021. The aim of this work was to support the ongoing implementation of the remote monitoring system.

**Methods:**

We used an action learning approach with rapid pragmatic methods, including informal discussions and observations as well as a feedback survey form designed based on the technology acceptance model to assess the use and acceptability of the system. Based on these results, we facilitated a meeting using user-centered design principles to explore user needs and ideas about its development in more detail.

**Results:**

In total, 21 users filled in the feedback form. The mean technology acceptance model scores ranged from 3.5 (for perceived ease of use) to 4.4 (for attitude) with behavioral intention (3.8) and perceived usefulness (4.2) scoring in between. Those working as nurses scored higher on perceived usefulness, attitude, and behavioral intention than did physicians. Based on informal discussions, we realized there was a mismatch between how we (ie, the research team) and the ward teams perceived the use and wider purpose of the technology.

**Conclusions:**

Designing and implementing the devices to be more nurse-centric from their introduction could have helped to increase their efficiency and use during the complex pandemic period.

## Introduction

The popularity of portable wearable technologies that monitor health has increased substantially over the past decade due to their perceived utility, relatively simple implementation, and immediate feedback [1]. Wearable technology is used in both personal and clinical settings, and more recently in the context of the COVID-19 pandemic for diagnosis, remote monitoring, and other applications in both inpatient and outpatient settings [1-3]. Using wearable devices for COVID-19 care can result in infection control by reducing the amount of time that health care workers (HCWs) are physically with patients and providing continuous monitoring of vital signs for the early identification and potential treatment of deteriorating patients [2]. Specifically, remote monitoring of oxygen saturation using wearable devices became increasingly common during COVID-19 in hospital settings [4,5].

Despite the potential benefits, there have been many challenges noted in implementing and using wearable devices during COVID-19, including technical, social, and political spheres [1]. Technical challenges often include battery life, Wi-Fi or Bluetooth connections, and device communication. A few examples of social challenges are patients lacking technological confidence (eg, in older patients) and repeated device alerts or continuous monitoring making patients nervous, while political challenges could include regulatory issues for expanding the approval of devices for COVID-19–related medical situations [1]. Many of these challenges may be enhanced in low- and middle-income countries (LMICs), while the need for such integration is crucial, especially during pandemic situations [6,7].

There are several studies exploring the technical challenges of integrating wearable devices in trial settings during COVID-19 (eg, see [8]), but there is a lack of research surrounding the acceptability of such devices within these contexts and how attitudes may impact actual use [9]. Portable wearable devices could be a potential solution to allow for continuous monitoring of vital signs remotely and affordably for COVID-19 wards in LMIC settings; however, while advantageous, these devices cannot meet their full potential if the users do not agree to use them or realize their potential value [10]. Understanding user perceptions and needs as well as the context in which the technology is implemented is crucial for successful implementation [1]. User-centered approaches stress the importance of integrating both human factors and technical factors [11] while also paying attention to avoid excluding certain populations in the design [7]. User-centered approaches have been cited as a “critical success factor” in a variety of health-related technology projects [12].

From August to December 2021, when COVID-19 cases were increasing more rapidly than at any time previously in Ho Chi Minh City, Vietnam, there was an opportunity to integrate a prototype wearable device and monitoring system into the COVID-19–designated wards at the Hospital for Tropical Diseases (HTD). At this time, the HTD was overwhelmed with patients with COVID-19 and we needed to deploy something urgently that could help. Using pragmatic methods during the rollout of the device, we describe stakeholders’ use of the wearable device, aspects of acceptability, and under which circumstances its use would be most beneficial for improving the care of patients with COVID-19. The primary objective of this work was to support the implementation process of the wearable device in the hospital to improve patient care during a catastrophic period of the COVID-19 pandemic in Ho Chi Minh City, Vietnam.

## Methods

### Study Setting

This work took place within a larger project called the Vietnam ICU Translation Application Laboratory (VITAL) at the Oxford University Clinical Research Unit (OUCRU) and HTD. The goal of VITAL is to design and implement innovative technologies to improve patient care within the intensive care unit (ICU) at the HTD, with a longer-term goal of expanding these technologies regionally. In addition to the clinical and technological studies, there is an ethnographic study to explore the sociotechnical contexts of the ICU at the HTD and within ICUs in Vietnam more broadly. The VITAL multidisciplinary team was in place at the start of the COVID-19 pandemic.

In the first 100 days of the COVID-19 pandemic, Vietnam rapidly implemented a variety of public health measures resulting in relatively few cases and zero deaths [13]. Since that time, there were a few concentrated outbreaks (for example, in Da Nang in July 2020 and December 2020 in northern Vietnam). In May 2021, the cases started to increase on a countrywide basis, and by August 2021, the hospitals began to fill with patients with COVID-19. It was within this pandemic context that the wearable device was implemented at the HTD, and the VITAL study teams worked together throughout to improve its implementation.

The wearable device was selected by the company and was already integrated into a locally developed platform based on an available application programming interface, licensing, and availability. The device was medical grade and measured heart rate and blood oxygen levels, similar to a pulse oximeter. The wearable device was battery powered and each one connected to a tablet that was kept at the patients’ bedside. The tablets had a 3G or 4G sim card and sent the data to a cloud where multiple patients’ data were viewable by HCWs outside the patients’ rooms and isolation area.

### Study Design

The aim of this work was to support the ongoing implementation of the wearable device rather than to follow a predefined, replicable study protocol, as would be used in trial settings, for example. Therefore, the work here describes the pragmatic rollout of the device. We used an action learning approach, including integration of multiple methods to assess the use and acceptability of the wearable device [14]. Action learning approaches rely on an iterative process of assessing local contexts, learning from relevant stakeholders, and using the information to improve an implementation or further develop a technology specific to the context [15,16]. As the wearable device started to be implemented in the HTD wards, our team of HCWs, social scientists, and technology developers took the opportunity to work together to inform the implementation. Therefore, we adapted the methods as the situation changed and more insights were gained [14].

### Participants

Potential participants included the HCWs from the HTD who were using the device in the wards during the implementation and corresponding ward heads. We estimated that a total of 30 doctors and 60 nurses would have worked in the wards where the wearable device was implemented and potentially used it in some form; therefore, we planned to recruit participants from this larger sample.

### Data Collection Methods

#### Informal Discussions and Observations

We used an iterative process of engaging in informal discussions coupled with sense-checking discussions and observations during the implementation period. The informal and sense-checking discussions and observations were conducted with the team who was working directly in the wards, as well as with head nurses from the wards where the wearable device was being implemented. The informal discussions and observations were conducted during the implementation of the device.

#### Feedback Survey Form

We created the feedback form based on the components of the technology acceptance model (TAM) to assess the use and acceptance of the device. The TAM is used in a variety of disciplines to determine how individuals accept (or not) and use (or not) a given technology. Davis [17] developed this model based on components from the theory of reasoned action [18] and it consists of the following variables: use motivation (with perceived ease of use and perceived usefulness) and behavioral intention [17,19]. The model suggests that an individual will accept the use of a technology (ie, their behavioral intention) based on their perception of the technology’s usefulness and ease of use. Perceived usefulness refers to the perception that using the technology will enhance one’s work; for example, the wearable device will provide physicians and nurses some advantages (eg, remote monitoring). Perceived ease of use refers to the perception that the use does not add more work or effort to the work that could be enhanced; for example, using the wearable device will not increase nurses’ workload, despite its utility and simplicity [17]. The TAM framework was expanded twice to include attitudes as well as several other external factors [20]. The use of the TAM in health research has shown how perceived usefulness and perceived ease of use relates positively to attitude and behavioral intention [21]. The TAM has been criticized for being insensitive to the context or social factors, being simplistic, and following an assumption that users are rational decision makers, when indeed other factors play into decision making [22-24]. We used the TAM framework for its simplicity and because the categories of perceived usefulness and perceived ease of use were of relevance, but we also integrated other data collection methods alongside it to counter these limitations to some extent.

Based on the components of the TAM, we included 23 questions related to usefulness (n=5), ease of use (n=5), attitude (n=5), and behavioral intent (n=8) [25]. We asked these questions using a 5-point Likert scale (with scores of 5 being more favorable). We also added 2 open-ended questions and collected a variety of relevant demographic information (Multimedia Appendix 1). We piloted the tool in both English and Vietnamese and adjusted the form as needed. We used Google forms for electronic self-completion of the form and offered paper forms for hand-written self-completion. We explained the feedback form to the ward staff during team meetings and provided the link. The feedback form was distributed and completed in Vietnamese. We kept the feedback form link open for 7 weeks in total and started data collection after the implementation had been integrated into the wards so that users would have had experience using the device.

#### User-Centered Workshop

We held a user-centered workshop with a selection of HTD ward staff to explore user needs and ideas for development in more detail. Because we already had the technology and knew the spaces where implementation would be held, we followed an adapted version of the process described by Cooper et al [26]. With this approach, the workshop participants and facilitators set the scene as a busy COVID-19 ward during the peak of the pandemic. Then, the facilitators described the shells of users (personas), including a nurse and a doctor persona shell, and we had the workshop attendees describe who they imagined the nurse and doctor to be, as well as their behaviors and needs and the values each user group would find most essential. We based the conversation on the wearable technology that the participants had already used. Then, the group discussed solutions to the issues identified [26].

### Data Analysis

Using the principles of action learning, we integrated the responses from informal discussions and observations into subsequent data collection, as well as summarized the content and grouped it into themes. For the analysis of the feedback survey form, we calculated mean scores for each variable and compared scores by profession. For the open-ended survey questions, we used content coding to summarize the responses topically. We presented the demographic data descriptively. We documented the responses from the user-centered design workshop as notes and summarized the results into main themes.

### Ethical Considerations

In this paper, we are describing the processes that occurred as part of the development and implementation of a monitoring system; therefore, the work did not require ethics approval. Prior to the initiation of any activities, we held a meeting with ward heads to describe the work in more detail and obtain their agreement.

## Results

### Device Implementation Within the HTD Context

The wearable device was implemented in 3 wards starting in August 2021, including the adult ICU, Ward A, and Ward E. We describe the implementation over a 5-month period from August to December 2021. During this period, these wards changed from COVID-19–designated and then back again to routine patient care settings, depending on the number of patients. Although the HTD was one of the COVID-19–designated hospitals, throughout the pandemic they offered routine patient care for specific diseases (eg, tetanus).

In addition to the rapidly changing physical spaces, the hospital management quickly deployed remote monitoring capacity using existing closed-circuit television cameras as a temporary solution to monitor very sick patients from outside the patients’ rooms. The remote monitoring was useful as it allowed for multitasking and prevented nurses and doctors from checking on patients more routinely in person. The hospital wards were at capacity during the study period. Prior to the pandemic, however, it was not unusual for the wards at the HTD to often be at maximum patient capacity. For example, in the adult ICU or during the rainy season, the number of dengue patients increases dramatically and the wards tend to be full.

Also, the workflow was organized differently during the pandemic period. Instead of nurses taking care of a few specific patients for the whole shift, 2 nurses and 1 doctor would instead go into the ward (in full personal protective equipment) as a team for 3 hours at a time while the other 2 nurses on shift completed admin work in the office. This meant that more coordination was needed, and often the team with the patients “need[ed] someone else to be [their] memory” as it was not easy to remember everything about all patients. The health care team’s workload, especially that of the nurses, ended up being more extensive for many reasons. One important reason is that, because of COVID-19 restrictions, there were also no families allowed in the wards who would help to look after patients in non–COVID-19 times; therefore, the majority of the care was left to the nurses. The patients were also more severely ill than previously in these wards and required more care by fewer staff.

### Device Use and Acceptability

When we first distributed the feedback form, out of 90 potential participants, only 22 completed the survey (19 electronic and 3 paper forms), and 1 person stated that they did not use the technology and therefore no responses were recorded for that participant. Of the 21 respondents who completed the feedback form, 48% (n=10) were doctors and 48% (n=10) were nurses, with 52% (n=11) of the participants coming from Ward E (Table 1).

Overall, when assessing the TAM variables, the mean (SD) scores ranged from 3.6 (0.8) for perceived ease of use to 4.4 (0.6) for attitude, with behavioral intention (mean 3.9, SD 0.6) and perceived usefulness (mean 4.2, SD 0.7) scoring in between. Those working as nurses scored higher on perceived usefulness, perceived ease of use, attitude, and behavioral intention than did physicians (Table 2).

When asked, as an open-ended question, why participants would or would not use the wearable device in the future, of the 19 responses inputted, 15 participants wrote that they would use the system because of its convenience and usefulness in monitoring patients. However, in 2 of those responses, they also added comments that the device had limited perceived accuracy and transmission problems. Of the remaining 4 participants, 1 participant simply stated that the monitor was still in use, 2 participants wrote that they did not use the system anymore due to job location changes, and 1 participant wrote a few sentences about why the wearable device is not the “best choice,” highlighting its limited battery life, how the system had become additional work for the already overworked staff, and how it is not yet completely implemented.

**Table 1:** Demographic characteristics of the survey respondents (n=21).

Characteristic		Value
**Gender, n (%)**		
	Women	13 (62)
	Men	8 (38)
Age (years), median (IQR)		35 (30-38)
**Occupation, n (%)**		
	Doctor	10 (48)
	Nurse	10 (48)
	Other: nurses’ aid	1 (5)
**Primary ward during the implementation phase, n (%)**		
	Adult intensive care unit	6 (29)
	Ward A	1 (5)
	Ward D	3 (14)
	Ward E	11 (52)

**Table 2:** Mean technology acceptance model (TAM) scores by variable. The maximum score was 5.

TAM variable	All participants, mean (SD)	Nurses, mean (SD)	Doctors, mean (SD)
Perceived usefulness	4.2 (0.7)	4.3 (0.8)	4.1 (0.6)
Perceived ease of use	3.6 (0.8)	3.8 (0.7)	3.4 (0.8)
Attitude	4.4 (0.6)	4.6 (0.6)	4.2 (0.6)
Behavioral intention	3.9 (0.6)	4.0 (0.6)	3.7 (0.7)

### Integrating User Perceptions for Improved Implementation

As part of the action learning process, we supplemented the feedback form results with data from the observations and informal discussions during the 5-month period. There were 3 main observations. First, there was a mismatch between how we (ie, the research team) and the ward teams perceived the use of the technology. We quickly realized, from our observations and from informal discussions with the implementation team, that many of the nurses either did not use the wearable device or did not think that they used it even if they used it in some aspect (eg, connecting the device for the patients or changing batteries). Even after we clarified what we meant by “use,” there were still not additional participants who filled in the feedback form because they felt like they did not use the technology.

Second, the ward teams had varying perceptions of the technologies that are routinely implemented by the OUCRU team in the HTD wards as part of research projects. We heard from informal discussions with colleagues that the nurses assumed the wearable devices were from a research project, as is often the case with OUCRU projects, and therefore the nurses, in particular, ignored the device even if they had some role in its use. They did not see its potential benefit.

Finally, in order to make the device more useful for the ward staff, we realized during the meetings and informal discussions with the team that we needed to make the implementation and use of the device more “pro-nurse,“ meaning we would need to emphasize how the device and its data were also useful and relevant to them. When discussing with the head nurse, the data were only displayed on the main screen in the staff room for one department. One suggestion was to move the tablet to the wall so that the nurses and others in the room (including the patients) could potentially see their vital signs. Because the devices and corresponding data were not in sight, it was easy to think that it was not relevant for the nurses and made it easier for them to ignore the device while with the patients.

### User-Centered Design Workshop

With the information we had learned from the informal discussions, observations, and feedback form, we held a follow-up workshop on January 17, 2022, to discuss how we could make better use of the technology in the wards in COVID-19 situations in the future. The attendees included 2 doctors (1 man and 1 woman) and 3 nurses (2 women and 1 man). The participants discussed the behaviors and needs of the nurse and doctor persona. For both roles, the needs centered on having equipment and improved coordination. The nurses also mentioned more training needs, while the doctors’ needs were about the accuracy of monitoring (Textboxes 1 and 2).

There were 3 main value prop themes, including medical, technical, and patient themes. For medical aspects, the attendees discussed how the device should be able to provide highly accurate data, with appropriate alarms and cut-offs. For the technical theme, the device and software should be simple to connect and use, with a long battery life and stable connections during charging or switching devices. The display should be large and clear, and the data should be stored for a long period of time (ie, 7-10 days). Finally, for the patient theme, the device should be comfortable for the patients to wear to avoid them removing it.

There were several solutions discussed in the group to improve the use and efficacy of the wearable device (Table 3). Solutions included improving the credibility of the data, ideas to improve the ease of use, ways to make the alarms more consistent, and ideas for more ideal placement. One very specific issue that the group mentioned was that the alarms went off too much on the large display and the alarms were always red or black and blinking, and it was difficult to know if the device was turned off (due to patient discharge) or actually disconnected, which would require an intervention. The solution was to refresh the devices; however, if the alarms were excessive and not always indicating a real issue, trust in the device would remain low, so this was an important priority. They also suggested that the alarms and display on the tablet should be the same as the big screen, as they preferred screen consistency.

Another in-depth discussion was about moving the tablets to the walls and having the device plugged in all the time, which would solve the battery issues. They felt that the tablet could be set up on the wall but that brought up other issues about how to keep the device and watch safe after use. For some of the topics, the group used features of another wearable device that they had used in the wards in the past to inform their solutions (eg, device graphs and a line on the device for finger placement).

**Behaviors and needs of the nurses.**

Participant: Nurse Van is a 36-year-old woman. She is an administrative nurse and has a management job. She likes to have fun and has a family and 2 children. She is also responsible for bringing the kids to school and back.

Behaviors:

- Visit and provide direct patient care and monitor vital signs
- Carry out medical orders (ie, medications, blood tests, and nutrition)
- Assess, monitor, and hand over patients
- Work night duty
- Night shifts inform doctors on vital signs as prescribed

Needs:

- Equipment (eg, to measure blood pressure, temperature, oxygen levels, and heart rate)
- Training on diseases
- Teamwork and coordination

**Behaviors and needs of the doctors.**

Participant: Doctor Huong is a 30-year-old woman. She is flexible and very active. She is not married and has no children and currently lives in a hotel. She is on night shift every 4 nights, and at times she visits her home in another town in Ho Chi Minh City, which is far from the Hospital for Tropical Diseases.

Behaviors:

- Prescribe medications
- Update medical records
- Perform examinations and change treatments
- Data entry
- Check vital signs in patient rooms (with a portable monitor that they move around) for examination and to detect abnormalities

Needs:

- Equipment (eg, monitors)
- Coordination with nurses (progress: medical records)
- Re-evaluation and working with other doctors
- Accuracy of vital sign monitoring

**Table 3:** Solutions for improvement.

Topic	Specific solution
Data credibility	Adding a graph for signal strength
Ease of use	Adding a finger placement mark on the device Increasing the font size on the watch and tablet Tablets should be fixed on the wall
Alarms	Reduce the alarm colors and blinking on the screen Use the same display on the screen and the tablets for consistency Refresh the tablets for more accurate alarms
Battery issues	Keep the tablet plugged in
Device placement	Placement on the wall (but only with an increase in font size)

## Discussion

The HTD and OUCRU teams, along with the technology company, rolled out the wearable device in an extremely complex pandemic situation with a prototype system. In the end, the team used the device on over 100 patients. We assessed the usability and acceptance of the device over the implementation period when COVID-19 cases were peaking in the hospital and into the period when the COVID-19 cases were reducing. Similar to the literature on the topics, we found that the importance of understanding the users and their experiences using the device was crucial to get the most use out of these technologies.

There was a mismatch between our perception of who was using the device and those who thought that they were using or benefiting from the device on the ground. From the start, the device was designed and set up with doctors in mind, but in practice, the nurses’ roles and use were overlooked, even though they could also routinely use and benefit from the device. In our study, we found that the nurses who filled in the feedback form, on average, had slightly higher scores on 3 of the 4 TAM domains (ie, perceived usefulness, attitude, and behavioral intention), while the doctors, on average, scored the perceived ease of use slightly higher than the nurses. We know from the challenges with acquiring feedback that many nurses did not feel that they used the device even though they had some role in the device set-up and monitoring. Designing the device to be more nurse-centric from the early phases could have helped to increase the efficiency and definition of who is meant to use it. In the future, it is important to consider that the way the device is used might be dependent on the form of its use (eg, for triage, use in a pandemic emergency, or routine hospital use). We recommend the involvement of staff who could benefit from the technology, especially nurses in the hospital context, in the full implementation process. This could help to avoid mismatches in the perceptions of who the users are and who could and should benefit from the new technology. Research on integrating wearable devices during COVID-19 in Singapore also highlighted that device simplicity would encourage its use and the importance of making the technology fit into the current environment while not increasing or disrupting workflows [27].

The trust in the device and its data was an issue brought up several times during the implementation and feedback sessions. There are a variety of potential explanations for inconsistent data (eg, incorrect device placement or averaging of data); however, it reduced the credibility of the device for both doctors and, importantly, nurses. Data concerns about technology in clinic settings has been noted in other studies. For example, Faria et al [28] found that study clinicians reported that 36% of the data from a remote monitoring project were “invalid” for a variety of reasons, including low literacy of the patients and complexity of the device. Involvement from users from the beginning of the design and implementation process is crucial for design purposes but also to build trust and confidence in the devices [11]. While this project took place during COVID-19, which is a very specific circumstance, the broader findings resonate with research conducted prior to COVID-19 that focused on the implementation and scaling up of digital health technologies in LMICs. The recommendations also included integration of end-user feedback and engagement with all stakeholders throughout the design and implementation process [12]

There are limitations to this work. First, we did not collect data on the clinical worth or the accuracy of the data transmitted from the devices. Second, we focused on feedback from only heath care staff (ie, doctors and nurses), and from only a subset of those who perceived that they used the device, which may have excluded some users and limited the overall sample size. We did not include patients who could also inform device acceptance, especially if used in noncritical cases where patients are moving around and conscious. Finally, the implementation setting for this work is not typical of other hospital settings in Vietnam or possibly other LMICs, as the HTD is a large referral hospital with an international research institute attached to it.

In anticipation of future (novel) pandemic situations or integration of wearable technologies into a range of clinical settings more broadly, it is important to fully understand if and how the wearable devices could be used more effectively by doctors, and importantly, nurses in the wards, for monitoring of deteriorating patients, especially in LMICs where resources are already stretched. Using an action learning approach during the implementation process highlights the importance of integrating user perspectives, ideas, and solutions into development and design.

## Appendix

Multimedia Appendix 1 Study tools in English and Vietnamese.

## Abbreviations

HCW: health care worker
HTD: Hospital for Tropical Diseases
ICU: intensive care unit
LMIC: low- and middle-income country
OUCRU: Oxford University Clinical Research Unit
TAM: technology acceptance model
VITAL: Vietnam ICU Translation Application Laboratory
