# Supplementary material for: Acceptance and User Experiences of a Wearable Device for the Management of Hospitalized Patients in COVID-19–Designated Wards in Ho Chi Minh City, Vietnam: Action Learning Project
Source: JMIR Hum Factors. 2024 Jan 5;11:e44619. doi: 10.2196/44619 (PMC10773555; doi:10.2196/44619)
Supplement: Multimedia Appendix 1 [file humanfactors_v11i1e44619_app1.pdf]

Iparamed feedback survey

You are being asked to provide your opinions and feedback on the Iparamed technology that was introduced at HTD in August 2021. We are in the process of developing the technology and would like to make improvements based on your experiences with the system over the past months. We are interested to learn from a variety of stakeholders who have varying levels of engagement with Iparamed system. Your feedback will be used to inform future development of similar technology and its implementation. The feedback responses will not be linked to you individually.

On average, it takes 3 minutes to complete the survey.

Part 1. Iparamed Vital Signs Monitoring System

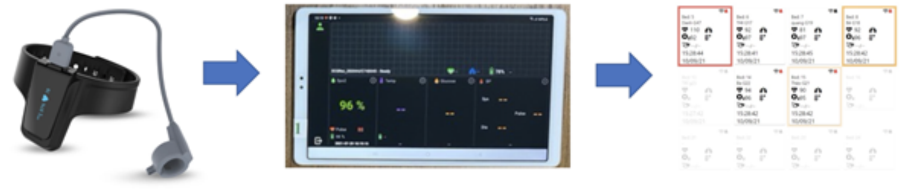

Did you work in an HTD ward where the Iparamed Vital Signs Monitoring System (see above image) was implemented?

☐ Yes (continue to Part 2) ☐ No (End the survery)

Part 2. Demographic Information

1. Please indicate the ward where you primarily work during August - October 2021

○ Ward A ○ Ward E ○ AICU ○ Ward D ○ Other, specify…………

2. Please mark all statements that are relevant to you: [tick all that apply]

☐ I check pulse and SpO2 data on the TV screen

☐ I select the patients who will use Iparamed system

☐ I connect the devices to patients

☐ I use data from Iparamed system for clinical decision making

☐ I maintain the Iparamed system (e.g. setting up, connecting, charging, cleaning, etc.)

☐ I manage the devices

☐ Other, specify

3. What is your age?

----

4. What is your gender?

○ Male ○ Female ○ Prefer not to say

5. What is your profession?

○ Doctor ○ Nurse ○ Other, specify

Part 3. Iparamed technology Section

For the following questions, please indicate your agreement with each statement using the scale from 1-5, with ***1 meaning strongly disagree*** and ***5 meaning strongly agree***.

	**Strongly Disagree**	**Disagree**	**Neutral**	**Agree**	**Strongly Agree**
Using Iparamed will improve the quality of monitoring patients (pulse, SpO2)	○	○	○	○	○
Using Iparamed will make my work more convenient	○	○	○	○	○
Using Iparamed will make me more effective in my work	○	○	○	○	○
Overall, I find the Iparamed to be useful	○	○	○	○	○
It's better to have Iparamed than no other monitoring equipment	○	○	○	○	○
It takes a lot of staff time and effort to set up and maintain the Iparamed system	○	○	○	○	○
I find the latest version of the Iparamed data screen to be clear and understandable	○	○	○	○	○
I found it easy to use the Iparamed system to make decisions about patient care	○	○	○	○	○
The Iparamed system collects patient vital signs data easily (pulse, SpO2)	○	○	○	○	○
Overall, I find the Iparamed system to be extremely difficult to use	○	○	○	○	○
I think Iparamed is a good idea	○	○	○	○	○
I think using the Iparamed is beneficial to doctors	○	○	○	○	○
I think using the Iparamed is beneficial for patients	○	○	○	○	○
I think Iparamed is beneficial for nurses	○	○	○	○	○
I have a positive perception of using the Iparamed	○	○	○	○	○
If available, I would use the Iparamed system with COVID-19 patients to quickly detect patients who are most sick	○	○	○	○	○

If available, I would use the Iparamed system for all patients admitted with COVID-19	○	○	○	○	○
a. If available, I would use the Iparamed system for **mild** patients admitted with COVID-19	○	○	○	○	○
b. If available, I would use the Iparamed system for **moderate** patients admitted with COVID-19	○	○	○	○	○
c. If available, I would use the Iparamed system for **severe** patients admitted with COVID-19	○	○	○	○	○
If available, I would always try to use the Iparamed system with all the patients in the wards (both COVID-19 patients and other patients)	○	○	○	○	○
If available, I would use the Iparamed system on every shift	○	○	○	○	○
I would prefer not to use Iparamed for vital signs monitoring (pulse, SpO2)	○	○	○	○	○

Have any patients provided comments on the Iparamed system to measure pulse and SpO2?

…………………………………………………………………………..

Please explain why you would or would not use the Iparamed technology in future.

…………………………………………………………………………..

Please provide any further comments on the Iparamed technology.

…………………………………………………………………………

Thank you for your time and feedback!

Khảo sát phản hồi hệ thống Iparamed

Chúng tôi gửi khảo sát này để xin ý kiến và phản hồi của bạn về công nghệ Iparamed đã được giới thiệu tại Bệnh viện bệnh nhiệt đới vào tháng 8 năm 2021. Chúng tôi đang trong quá trình phát triển công nghệ và muốn cải tiến dựa trên trải nghiệm của bạn với hệ thống trong những tháng qua. Chúng tôi muốn học hỏi từ nhiều bên liên quan có mức độ tham gia khác nhau với hệ thống Iparamed. Phản hồi của bạn sẽ được sử dụng để định hướng cho việc phát triển và triển khai của công nghệ tương tự trong tương lai. Các phản hồi sẽ không gắn với thông tin cá nhân của bạn.

Trung bình, mất khoảng 3 phút để hoàn thành khảo sát.

Phần 1. Hệ thống theo dõi sinh hiệu Iparamed

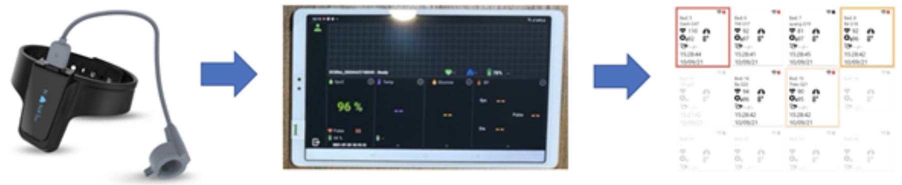

Bạn có làm việc ở các khoa của BVBNĐ nơi có triển khai hệ thống theo dõi sinh hiệu Iparamed (xem hình bên trên)?

☐ Có (chuyển đến Phần 2)　　☐ Không (Kết thúc khảo sát)

Phần 2. Thông tin nhân khẩu học

1. Vui lòng cho biết bạn làm việc ở khoa nào trong thời gian từ tháng 8-10/2021

○ Nhiễm A　○ Nhiễm E　○ HSCCNL　○ Nhiễm D　○ Khác, cụ thể…………

2 Vui lòng chọn tất cả những câu liên quan đến bạn đối với hệ thống Iparamed

☐ Tôi xem thông tin mạch và SpO2 trên màn hình TV

☐ Tôi chọn người bệnh để sử dụng hệ thống Iparamed

☐ Tôi gắn thiết bị vào người bệnh

☐ Tôi sử dụng dữ liệu từ Iparamed để đưa ra quyết định lâm sàng

☐ Tôi bảo trì hệ thống Iparamed (Vd: cài đặt, kết nối, sạc pin, vệ sinh..)

☐ Tôi quản lý thiết bị

☐ Khác, cụ thể

3. Bạn bao nhiêu tuổi?

----

4. Giới tính?

○ Nam ○ Nữ ○ Không muốn nói

5. Nghề nghiệp của bạn?

○ Bác sĩ ○ Điều dưỡng ○ Khác, cụ thể…………

Phần 3. Công nghệ Iparamed

Với các câu hỏi sau, hãy cho biết mức độ đồng ý của bạn với thang điểm từ 1-5 với ***1 nghĩa là hoàn toàn không đồng ý*** và ***5 nghĩa là hoàn toàn đồng ý***.

	**Hoàn toàn không đồng ý**	**Không đồng ý**	**Không ý kiến**	**Đồng ý**	**Hoàn toàn đồng ý**
Sử dụng Iparamed sẽ cải thiện việc theo dõi mạch và SpO2 của người bệnh	○	○	○	○	○
Sử dụng Iparamed sẽ làm công việc của tôi tiện hơn	○	○	○	○	○
Sử dụng Iparamed sẽ giúp giảm bớt khối lượng công việc của tôi.	○	○	○	○	○
Nói chung, tôi thấy Iparamed hữu ích.	○	○	○	○	○
Có Iparamed thì sẽ tốt hơn khi không có thiết bị theo dõi nào cả	○	○	○	○	○
Mất nhiều thời gian và nỗ lực của nhân viên để cài đặt và duy trì hệ thống Iparamed	○	○	○	○	○
Tôi thấy phiên bản gần đây của Iparamed trên màn hình TV rõ ràng và dễ hiểu.	○	○	○	○	○
Tôi thấy dễ sử dụng hệ thống Iparamed để đưa ra quyết định về chăm sóc người bệnh.	○	○	○	○	○
Hệ thống Iparamed thu thập thông tin mạch và SpO2 của người bệnh dễ dàng.	○	○	○	○	○
Nói chung, tôi thấy hệ thống Iparamed cực kỳ khó sử dụng.	○	○	○	○	○
Tôi nghĩ hệ thống Iparamed là một ý tưởng tốt.	○	○	○	○	○
Tôi nghĩ hệ thống Iparamed hữu ích cho bác sĩ.	○	○	○	○	○
Tôi nghĩ hệ thống Iparamed hữu ích cho người bệnh.	○	○	○	○	○
Tôi nghĩ hệ thống Iparamed hữu ích cho điều dưỡng.	○	○	○	○	○
Tôi có cảm nhận tích cực về hệ thống Iparamed.	○	○	○	○	○
Nếu có, tôi sẽ sử dụng hệ thống Iparamed cho người bệnh nhiễm COVID-19 để ưu tiên và phân bổ nguồn lực cho người bệnh nặng nhất	○	○	○	○	○

Nếu có, tôi sẽ sử dụng hệ thống Iparamed cho tất cả người bệnh nhiễm COVID-19 nhập viện	○	○	○	○	○
a. Nếu có, tôi sẽ sử dụng hệ thống Iparamed cho những người bệnh nhiễm COVID-19 nhẹ nhập viện	○	○	○	○	○
b. Nếu có, tôi sẽ sử dụng hệ thống Iparamed cho những người bệnh nhiễm COVID-19 trung bình nhập viện	○	○	○	○	○
c. Nếu có, tôi sẽ sử dụng hệ thống Iparamed cho những người bệnh nhiễm COVID-19 nặng nhập viện	○	○	○	○	○
Nếu có, tôi sẽ luôn sử dụng hệ thống Iparamed cho tất cả người bệnh ở các khoa (cả người nhiễm COVID-19 và người bệnh khác)	○	○	○	○	○
Nếu có, tôi sẽ sử dụng hệ thống Iparamed cho mỗi ca trực	○	○	○	○	○
Tôi sẽ không muốn sử dụng hệ thống Iparamed để đo mạch và SpO2	○	○	○	○	○

Người bệnh có ý kiến gì về hệ thống Iparamed để đo mạch và SpO2?

……………………………………………………………………………..

Vui lòng giải thích tại sao bạn sử dụng hoặc không sử dụng hệ thống Iparamed trong tương lai.

……………………………………………………………………………..

Vui lòng cho thêm ý kiến về hệ thống Iparamed.

……………………………………………………………………………

Chân thành cảm ơn bạn đã dành thời gian gửi phản hồi!
